# A call for using natural compounds in the development of new antimalarial treatments – an introduction

**DOI:** 10.1186/1475-2875-10-S1-S1

**Published:** 2011-03-15

**Authors:** Hagai Ginsburg, Eric Deharo

**Affiliations:** 1 Dept. Biol. Chem., Inst. Life Sci., The Hebrew University of Jerusalem, Jerusalem 91904, Israel; 2 Laboratoire de Pharmacochimie des Substances Naturelles et Pharmacophores Redox, UMR 152, UPS, Université de Toulouse, 118 Route de Narbonne, F-31062 Toulouse Cedex 9, France; 3 Institut de Recherche pour le Développement, UMR 152, Mission IRD Casilla 18-1209 Lima, Peru

## Abstract

Natural compounds, mostly from plants, have been the mainstay of traditional medicine for thousands of years. They have also been the source of lead compounds for modern medicine, but the extent of mining of natural compounds for such leads decreased during the second half of the 20^th^ century. The advantage of natural compounds for the development of drugs derives from their innate affinity for biological receptors. Natural compounds have provided the best anti-malarials known to date. Recent surveys have identified many extracts of various organisms (mostly plants) as having antiplasmodial activity. Huge libraries of fractionated natural compounds have been screened with impressive hit rates. Importantly, many cases are known where the crude biological extract is more efficient pharmacologically than the most active purified compound from this extract. This could be due to synergism with other compounds present in the extract, that as such have no pharmacological activity. Indeed, such compounds are best screened by cell-based assay where all potential targets in the cell are probed and possible synergies identified. Traditional medicine uses crude extracts. These have often been shown to provide many concoctions that deal better with the overall disease condition than with the causative agent itself. Traditional medicines are used by ~80 % of Africans as a first response to ailment. Many of the traditional medicines have demonstrable anti-plasmodial activities. It is suggested that rigorous evaluation of traditional medicines involving controlled clinical trials in parallel with agronomical development for more reproducible levels of active compounds could improve the availability of drugs at an acceptable cost and a source of income in malaria endemic countries.

## Background

Forty percent of the world’s population are exposed to malaria and there is a constant need for new anti-malarials in the face of the ever-present and ever-emerging resistance of parasites to currently available drugs, whether used in monotherapy or in combination. When the genome of *Plasmodium falciparum* was first deciphered, there were great hopes of finding new targets for drug development. Today, eight years later, these hopes by no means fully materialized. Although dozens of target-oriented compounds were shown to be anti-plasmodial in the nanomolar range, very few of them have reached the pre-clinical test phase [1,2]. Hence, it seems that new approaches for the development of antimalarial drugs should be considered, or old ones revisited. This Introduction argues for the second of these alternatives and to consider the potential of natural compounds for this quest.

## Natural compounds were historically used as drugs

Plants have been used as a source of medicine throughout history and continue to serve as the basis for many pharmaceuticals used today. The medicinal properties of plants are described already on Assyrian clay tablets dated about 2000 B.C. and documented in the Egyptian culture, the Indian Ayurveda [3], in Traditional Chinese Medicine (TCM)[4], and various European documents. Traditional medicines not only provide valuable clues for finding new drugs, but also may help to shift the drug discovery paradigm from ‘finding new-entity drugs’ to ‘combining existing agents’, and might even direct the combinations between such agents. [4,5]. A recent structural comparison between ~10,000 traditional TCM components and ~8,000 modern drugs or candidates, identified 908 agent pairs that are structurally similar (with similarity 0.85) and 327 agent pairs that are identical in structure. Plants continue to serve as the basis for many pharmaceuticals used today [6]. Although the modern pharmaceutical industry was born from botanical medicine, synthetic approaches to drug discovery have more recently become standard. Although a comprehensive review of human drugs introduced between 1981 and 2006 indicates that about 62 % of new small-molecule drugs were either natural products, derived from natural products (usually semi-synthetically) and natural product–inspired pharmacophores (that could be considered natural-product analogs) [6], the synthetic combinatorial chemistry and high throughput screening (HTS) of potential drug targets disconnected the historical link between plants and medicines. Today, however, the small output of modern antimalarial pharmaceutical research and development has stimulated new interest in the potential of natural compounds.

In the pharmaceutical industry in the 1990s, the high-throughput screening of chemical libraries against potential targets was emphasized while, in parallel, screening of natural product diminished. The transition from natural sources to combinatorial libraries subjected to high throughput screening (HTS), seem to be have been based on the apparent advantage of rational drug design versus “random” screening. There was perhaps also a feeling that ethnobotany was a “soft science” and not a scientifically valid paradigm for the discovery of new drug leads.

## Why are plants a prolific source for drugs?

One can make an argument that biologically-derived secondary metabolites and synthetic compounds derived from them perform better as drugs than do randomly synthesized compounds. Primary and secondary metabolites, receptors, enzymes, transporters and regulatory proteins originated from a limited number of parent molecules. These originating molecules were present in primitive life forms and therefore co-evolved to interact with one another, thus granting direct ecological benefit to the producing organism, whether in competition for resources, avoiding predation or combating pathogens. Although a divergence in function and structure has subsequently occurred, some structural relationship still remains that make natural products, on average, better ligands for biological targets than randomly synthesized compounds [7,8]. Biosynthesis uses a very parsimonious set of building blocks. It diversifies by taking its limited battery of building blocks and distributes them into many different pathways. Nature has a tendency to form oxides by hydrolysis or abstraction of oxygen from organic compounds by enzymes that delicately achieve site-selective C–H activation to introduce oxygen and discriminate between numerous functional groups at different oxidation levels. In contrast, medicinal chemistry focuses on nitrogen and often includes additional atoms such as sulfur and halogens that are relatively rare in nature [9]. Generally, natural metabolites have a high ‘sterical complexity’. This is an evident outcome of the spatial dimensionality and the chirality of the enzymes involved in biosynthesis, their molecular targets and the metabolites themselves [10]. Bioactive compounds share pharmacophoric features, and natural products provide a rich source of potentially attractive scaffolds and molecular building blocks for synthesis. Since natural compounds have not evolved as therapeutic agents, sometimes their chemical structure must be further improved, whether in terms of efficacy and selectivity for the target or achieving optimal pharmacokinetic and pharmacodynamic properties. The numerous ring system scaffolds have no orthologs in the drug synthesis database. Hence, natural products could provide new starting points in drug discovery. The vastly unexplored flora and fauna (90 % of total species) could provide other new leads and drugs for chemotherapy, for the isolation of certain natural products in large amounts, total synthesis by chemical approaches, or limited scope for chemical modification.

## Natural compounds should be used in cell-based assays

One of the reasons for the relative failure of modern medicine R&D in the malaria field, may be due to technological shortcomings. While target-oriented HTS is valid for the screening of libraries of synthetic and/or natural compounds, the underlying biochemical assays are sometimes too sensitive and too prone to artifacts. This arises because of issues of solubility, aggregation, chemical reactivity and quenching effects, which detract from the effective screening of *natural extract* mixtures. To overcome these difficulties it seems preferable to use functional biologic assays on pathogens or their host cells, not biochemical assays on a purported target. In the cell-based assay, all the potential targets in the cell are screened by all compounds present in the extract (see below for deeper discussion). There are nevertheless several limitations facing the development of drugs from natural sources. To name but few, many compounds cannot be further developed because of their toxicity, low bioavailability and/or poor solubility. Isolation of certain natural products in large amounts may be a limitation as is the limited degree for chemical modification. The loss of plant species and habitats through environmental change is a worry, while agriculture may be a limitation in some cases when yields are low or when growth periods are long. Large-scale harvesting of medicinal plants from forests may affect the forest ecology or even a total extinction of the prospected species.

## Synergy between constituents of crude extracts

A major difficulty in finding single-component new-entity drugs from natural medicines originates from the fact that the efficacy of most natural medicines may lie in the synergy or additivity of diverse components rather than arising from a single compound. [11,12] The synergy of natural medicines arises from the co-evolution between l plants and their foes [13]. For example, plants developed antimicrobials to fight against the invasion of diverse pathogens. To survive the antibiotic assault, pathogens evolved resistant systems, such as MDR pumps. In the next round of the arms race, plants were stimulated to develop MDR inhibitors [14]. Thus, a crude extract can contain both active inhibitors and their potentiators [15]. Additive and synergistic effects are subsets of the pharmacodynamic of potentiation, where different compounds in a mixture interact to provide a combined effect that is equal to the sum of the effects of the individual components (additive), or where combinations of bioactive substances exert effects that are greater than the sum of individual components (synergistic). Potentiation can exist between different phytochemicals within a single plant extract or with different plants or between a phytochemical and a synthetic drug. Drug combinations may produce pharmacokinetically synergistic or antagonistic effects such that a potentiator could act by regulating the absorption, distribution, metabolism and excretion of the therapeutically active drug. While pharmacodynamic interactions which occur at the cellular level can be tested *in vitro* in cell assays, pharmacokinetic interactions can be identified only *in vivo*. Although not a very frequent observation, cases are reported where all the compounds found in an extract are inactive individually but are pharmaceutically active in combination (see Deharo and Ginsburg and Rasoanaivo *et al*,, this issue). The more common observation is the potentiation of one active ingredient by another inactive component. The tea prepared with *Artemisia annua* on the dosage recommendations of the current pharmacopoeia of Chinese medicine contains in total 94.5 mg/l of artemisinin, which is only 20 % of the usually recommended daily dose in conventional treatment. In conformity with this, it was demonstrated that of the 36 flavonoids produced by *A. annua*, five (artemetin, casticin, chrysoplenetin, cirsilineol and chrysosplenol-D), potentiate the *in vitro* activity of artemisinin against *P. falciparum*, while having almost no activity on their own [16-18]

Experimental studies have shown synergy between the various Cinchona alkaloids (quinidine, cinchonine and cinchonidine, all found in crude extracts of the Cinchona bark) with improved activity over quinine against resistant *P. falciparum* in vitro [19], particularly when quinine is combined with cinchonine [20].

In general, synergistic and potentiative drug combinations have been shown to achieve one or more favorable results: enhanced efficacy, decreased dosage at equal or increased level of target inhibition, reduced or delayed development of drug resistance and simultaneous reduction of toxic effects. The mechanism of many of these activities can be understood at the cellular level by using network and system analyses [13]. Thus for example, synergy may arise from interactions with an anti-target or counter-target, from negative modulations of a network’s robustness or from compensatory and neutralizing actions. Synergy can also result from complementary actions which may involve positive regulation of a target or process by targeting multiple points of a pathway (such as in the case of sulphadoxine and pyrimethamine used for malaria chemotherapy, which act on 2 different targets of the folate biosynthesis pathway), interacting with multiple sites, states, conformations and mutant forms of the same target [21]. Drug combination in antimalarial chemotherapy is widely used [22]. It was adopted as a strategy to prevent the evolution of resistance against either component of the combination.

Importantly, it is not unlikely that the behavior of some extracts will be significantly altered by various interactions with the host’s metabolism and physiology, leading either to reinforcement or to the opposite. Developing innovative scientific methods for discovery, validation, characterization and standardization of such multicomponent botanical therapeutics is essential to their acceptance into mainstream medicine [23].

Before the 20th century therapy relied almost exclusively on multicomponent medicines, obtained from natural sources [24]. Presently, an increasing number of diseases are being treated with combinations of several single-component drugs with the aim to lower the evolution of resistance or to target several pathological processes simultaneously. They are used in treating infectious diseases (HIV, malaria and TB) and composite chronic diseases like cancer.

## Prospecting for natural compounds

The validation of traditional remedies can be problematic because of the lack of sufficient information, documentation and of standardization of extracts to be evaluated, but these remedies deserve deep and thorough consideration [25]. In spite of these problems, such materials serve as a valuable source for novel compounds. They also conceal an abundant combination of secondary metabolites which might act in concert to enhance the therapeutic effect [26,27]. Because the quantitative and qualitative composition of secondary metabolites in a plant is notoriously varied, standardization is obligatory. Modern agronomic and plant genetic methods could be harnessed to domesticate some of the most promising medicinal plants, so as to ensure a reproducible qualitative and quantitative production of active ingredients, in spite of changes in biotic and abiotic constraints. Achieving such goals may provide indigenous populations in malarious areas with new commercial crops. In addition, the road to antimalarial development could be shortened considerably, since it is much easier to approve the use of extracts than that of their active ingredients. This does not mean that the use of such extracts does not have to pass through rigorous clinical test to assess that toxicity, teratogenicity, mutagenicity etc are not a problem.

## Natural compounds as a source for antimalarial drugs

Research on natural compounds has already contributed to the discovery of new antimalarial drugs. Atovaquone, artemisinin and its semi-synthetic derivatives as well as clindamycin, erythromycin, azithromycin, chlortetracyline, tetracycline, oxytetracycline and doxycycline, are noteworthy examples of the varied contribution of natural products for the development of effective antimalarial drugs, particularly valuable for the treatment of chloroquine-resistant parasites. Several comprehensive reviews on the antimalarial potency of plant products derived from ethnic medicine were published in the last decade and are summarized in Table 1. The quality of the data used in these reviews differs and in most earlier reports the chemical structure of the purified compound was not known and toxicity tests were not performed. However, in many cases good activity and selectivity were observed. Most importantly, several compounds containing unique structural composition have been isolated and characterized. It is therefore not surprising that natural compounds dominate the recent malaria patent literature [28,29]. Although many compounds cannot be further developed for reasons mentioned above, the discovered lead compounds provide valuable bioactive scaffolds which could be further adjusted by semi-synthetic approaches to obtain effective anti-malarials [30] See also Wells and Guantai1 & Chibale, this issue.

**Table 1:** Anti-malarial compounds from plants- recent reviews

Source	Number of plant species or compounds	Nature of compound	Availability of chemical structure	Quantitative toxicity tests
Tgaboto and Townson 2001^1^[36]	270 species	extracts or purified components	No	No
Schwikkard and and van Heerden 2002^2^[37]	170 compounds	purified	Yes	No
Saxena et al 2003^3^[38]	250 compounds	purified	Yes	Few
Frederich et al, 2008^4^[39]	31 compounds	purified	Yes	vitro, vivo, good selectivity
Pillay et al, 2008^5^[40]	216 species and 24 compounds	extracts or purified components	For purified compounds	No
Batista et al, 2009^6^[41]	60 species	extracts or purified components	for some	vitro, vivo, many with good selectivity
Kaur et al, 2009^7^[42]	266 compounds	purified	Yes	vitro, vivo, many with good selectivity
Mariath et al, 2009^8^[43]	198 species	extracts	No	vitro, vivo
Bero et al, 2009^9^[44]	301 compounds	purified	Yes	vitro, vivo

1. Compiled a list of 270 plant species whose extracts or purified components have anti-malarial activity but no chemical structures or details about their potency were provided.

2. A total of 170 structures have been reviewed from 186 references found in the literature up to December 2000

3. Provides a critical account of crude extracts, essential oils and anti-plasmodial secondary metabolites with diverse chemical structures from higher plants, covering the period 1993-2003. A total of 127 alkaloids, 18 quassinoids, 23 sesquiterpenes, 27 triterpenoids, 21 flavonoids/xanthones, nine quinones and 25 miscellaneous compounds were highlighted in their work, although very few quantitative details are provided.

4. Three ethnobotanical screening programmes have been conducted on South African plants while there have been a few studies adopting a more direct approach, where plants within a particular genus were screened for anti-plasmodial activity. The paper also summarizes the bioactive molecules identified from selected plants having anti-plasmodial activity.

5. Covers 31 indole alkaloids isolated from natural sources with high anti-plasmodial activity (*in vitro* and *in vivo*), most of them displaying IC_50_ values under the micromolar range and with a good selectivity index.

6. Review anti-plasmodial non-alkaloids natural products from reports published in the period Jan/2008-May/2009. Compounds include the classes of terpenes, limonoids, flavonoids, chromones, xanthones, anthraquinones, and other miscellaneous and related compounds .The review covers 60 plant species belonging to 34 families, some of them extracted by 3 different solvents. Twenty four extracts were found with significant activity, e.g., IC_50_<3 μg/ml. Some were also tested *in vivo*. Most extracts show only weak activity in culture or in mouse models. Many recent reports on anti-plasmodial activity of plants used in local ethnic medicine are, however, not reviewed.

7. Review focusing on anti-plasmodial compounds discovered during 1998-2008 from all natural sources, including crude extracts from plant and marine organisms. A total of 266 anti-plasmodial natural products, for most the available chemical and pharmacological details are shown. The compounds listed in this compilation belong to the classes of alkaloids, terpenes, quassinoids, flavonoids, limonoids, chalcones, peptides, xanthones, quinones, coumarins and miscellaneous compounds, as well as 37 promising semisynthetic anti-malarials. The review also presents progress in recent semi-synthetic approaches to develop drugs from natural sources which display some anti-malarial activity. The semi-synthetic compounds belong to different classes: alkaloids – naphthylisoquinoline, bisbenzylisoquinoline, protoberberine, aporphine, indole alkaloids, manzamine alkaloids and others. Terpenes – sesquiterpenes, triterpenes, diterpenes and others. Other semi-synthetic compounds belong to quassinoids, flavonoids, limonoids, chalcones, peptides, xanthones, quinones and coumarins. For many of them, data on anti-plasmodial activity in culture and anti-malarial activity in mouse models and toxicity facts are provided. Most importantly, several compounds containing unique structural composition have been isolated and characterized.

8. Review plants of the American continent with anti-malarial activity, describing 198 plants whose extracts were active *in vitro* and *in vivo*. *In vivo* tests were done on *P. berghei*, *P. gallinaceum*, *P. vinckei*, *P. lophurae*, *P. cathemerium* and *P. yoelii* strains.

9. Review only compounds purified from plants used in traditional medicine, covering publications between 2005 and 2008. The list of compounds contains flavonoid derivatives, xanthones, coumarins, lignans, tannins, diterpenes and triterpenes, steroids, and derivatives of ornithine, lysine, phenylalanine, tyrosine and tryptophane derivatives, as well as steroidal alkaloids. Alkaloids and diterpenes are the most numerous among the highly active compounds (IC_50_ ≤2 μM), while coumarins, steroids, stilbenes and tannins provide only moderate activity (2 < IC_50_ ≤ 11 μM). The Caesalpiniaceae family provides the highest number of highly active compounds. But the Asteraceae, Leguminosae and Moraceae are significant contributors.

## Screening of natural compounds for antimalarial activity

All this said, it could have been expected that prospecting for drug leads in natural sources would systematically compare the biological activity of the extract in parallel to that of purified compounds thereof. This is not the case: The Eskitis Institute for Cell and Molecular Therapies has screened over 200,000 compounds purified from 40,000 samples of plants and animals collected from oceans and forests in Australia, Papua New Guinea and China for their anti-plasmodial activity, but has never compared their effect to that of the crude extract from which they were purified. The same practice is ongoing in other institutes and companies such as BioFocus DPI-UK, The University of South Florida, Harvard Medical School and Novartis Natural Products Unit. It is quite possible that many synergistic combinations may have been missed. A thorough search of the literature has revealed dozens of cases where the extract has a more potent anti-plasmodial activity compared to its most potent compound (see article by Deharo and Ginsburg in this issue). A revision of the screening strategy seems warranted.

## The prospective of traditional/ethnic medicine

In some Asian and African countries, 80% of the population depends on traditional medicine for primary health care[31]. However, this market is plagued by counterfeit, poor quality, or contaminated herbal products posing serious patient safety threats [**32**].

More than 100 countries have regulations for herbal medicines. Developing innovative scientific methods for discovery, validation, characterization and standardization of these multicomponent botanical therapeutics is essential to their acceptance into mainstream medicine. Validation is limited by lack of prioritization of plant species for research and absence of information on ethnobotany of these plants (location and abundance, parts used, form of use, duration of treatment), definition of dosages due to variation in the concentrations of active ingredients in a plant species. It should also be stressed that a basic requirement for the validation of a medicinal plant is the standardization of extracts to be evaluated, including the identification and quantification of chemical and/or biological markers to assure the development of efficient and safe phytomedicines in a short time and at low cost.

Testing the effects of extracts usually doesn’t divulge the drug target or its mode of action. But if the extract is working well is it really important as to know the precise mode of action? In the context of anti-malarials, it is relevant to underscore the fact that till this very day, the mechanism of action of two of the most efficient drugs derived from traditional medicine, artemisinin and quinine, is still debatable. A search in PubMed for “traditional/ethnic and plant and malaria” retrieves 293 publications, implying that traditional herbal medicines are a valuable source for drug development. But shouldn’t one reconsider the utilization of ethnic/traditional medicine as such since it integrates active compounds and their combinations? Regulating traditional medicine products, practices and practitioners is difficult due to variations in definitions and categorizations of traditional medicine therapies. Evidence from clinical tests done to evaluate the safety and effectiveness of traditional medicine products and practices is limited. Requirements and methods for research and evaluation are complex. For example, it can be difficult to assess the quality of finished herbal products as the safety, effectiveness and quality of the final product depends on the quality of their source materials and the control of the production processes. The quality and uniformity of the chemical constituents and their concentrations in natural products may be affected by factors such as subspecies or age of plant, geographical and seasonal variations, time and method of collection and storage, etc. Expanding the herbal materials market for products collected from wild plant populations and cultivated medicinal plants could drive over-harvesting of plants and threaten biodiversity. Efforts to preserve both plant populations and knowledge on how to use them for medicinal purposes is needed to sustain traditional medicine. In addition, domestication of prioritized plants in combination with modern genetic engineering and agro technological methods, could assure a sustained and reliable source of material for herbal medicine that will contain the desired and expected amounts of pharmacologically active constituents and their potentiators. Achieving such goals could also provide additional means of production to indigenous countries and self sufficiency in antimalarial drugs when the present high level of international support in the purchase and distribution of modern drugs will dwindle with time.

## How the use of traditional medicines is being considered in practice?

WHO and its Member States are already cooperating to promote the use of traditional medicine. The goals of this collaboration are to support and integrate traditional medicine into national health systems in combination with national policy and regulation for products, practices and providers; to ensure the safety and the quality of the products and practices, based on available evidence; to acknowledge traditional medicine as part of primary health care; to preserve knowledge and resources ensure patient safety by upgrading the skills and knowledge of traditional medicine providers. What seem to be overlooked, yet it should be very high on the agenda, is the development of rigorous protocols for the clinical testing of traditional remedies. Such tests should consider anti-disease efficacy, toxicity, mutagenicity, age- and pregnancy-dependent effects, antagonism or synergism with presently used anti-malarials and survey of possible evolution of resistance.

In parallel, the African Network for Drugs and Diagnostics Innovation (ANDI) has been recently launched in Abuja in 2008 and established at the 2nd ANDI Stakeholders' Meeting in 2009 in Cape Town, with the mission to promote and sustain African-led health product innovation to address African public health needs through the assembly of research networks and building of capacity to support human and economic development [33]. Health product innovation as used here covers the entire product R&D value chain, including new drugs based on traditional medicines and natural products. Hopefully, these initiatives will result in the development of indigenous means for chemotherapy and prophylaxis of malaria at an affordable price and sustainable efficacy. Relevant to this hope is the recently updated FDA regulations for multicomponent botanical therapeutics (“Guidance for Industry: Botanical Drug Products,” published by the FDA Center for Drug Evaluation and Research in 2006 [34,35]). The guidance provides an exemption for botanical drugs with some prior history of human use, allowing them to advance through phase II clinical trials with fewer preclinical and toxicological studies than would be required for new chemical entities. In addition, the guidance does not require full characterization of all extract components or full elucidation of their interactions, and may tolerate some variation in the final composition of the botanical drug.

## Conclusions

In this short essay, the rationale for using natural compounds for drug development has been presented with specific consideration of anti-malarial drugs. The underlying principles for using drug combination and the benefits of using multi-component plant extracts have been argued. It seems that the scientific and pharmacological justification of these principles could provide the basis for further development of ethnic/traditional medicine as a valid, cheap and locally-available means to treat malaria.

## Competing interests

The authors declare that they have no competing interests.
